# On the influence of inhibitory STDP on balanced state random networks

**DOI:** 10.1186/1471-2202-14-S1-P200

**Published:** 2013-07-08

**Authors:** Felix Effenberger, Anna Levina, Jürgen Jost

**Affiliations:** 1 Max-Planck-Institute for Mathematics in the Sciences, Leipzig, 04103, Germany; 2 Bernstein Center for Computational Neuroscience Göttingen, 37077, Germany

## 

The distribution of synaptic efficacies in neural networks takes fundamental influence on their dynamics and the modification of synaptic strengths forms the foundation of learning and memory. A prominent plasticity rule that has been observed in vitro is spike-timing-dependent plasticity (STDP). While first studied in glutamatergic synapses, recently also STDP of GABAergic synapses came into the focus of experimental and theoretical research [1].

We study random balanced state networks of leaky integrate-and-fire neurons in the asynchronous irregular (AI) regime [2] that is believed to be a good theoretical fit to the activity of cortical networks in vivo. We consider driven networks that receive Poisson input as well as networks in a self-sustained state of activity. In order to assess the influence of excitatory and inhibitory STDP on the network dynamics, we introduce these two plasticity rules independently, observing network dynamics and weight distributions after a transient phase. Note that both additive and multiplicative STDP rules yield the same network dynamics as described below.

When introducing excitatory STDP alone, parameters involving the maximal weight have to be fine-tuned in order to keep the network activity stably in the AI regime [3]. For almost all parameter values the network activity becomes unstable, leaving the AI regime and settling in a pathological, highly synchronized state with saturated firing rates of most cells, see Figure 1A. We also observed that even without STDP, few strong excitatory connections can substantially destabilize network dynamics yielding pathological states. Interestingly, this destabilization does not happen when in addition to excitatory STDP we also introduce STDP for inhibitory synapses projecting onto excitatory cells. The latter setup results in a network that stably rests in the AI regime, see Figure 1A. Both STDP rules yield near-Gaussian distributions of synaptic weights, see Figure 1B. Inhibitory STDP even manages to stabilize a network that was brought to a pathological state by excitatory STDP, see Figure 1A. This clearly shows that inhibitory STDP has a stabilizing effect on network dynamics and we expect that especially in combination with synaptic scaling and in the context of clustered networks [4] other non-trivial dynamical effects will become visible.

**Figure 1:**
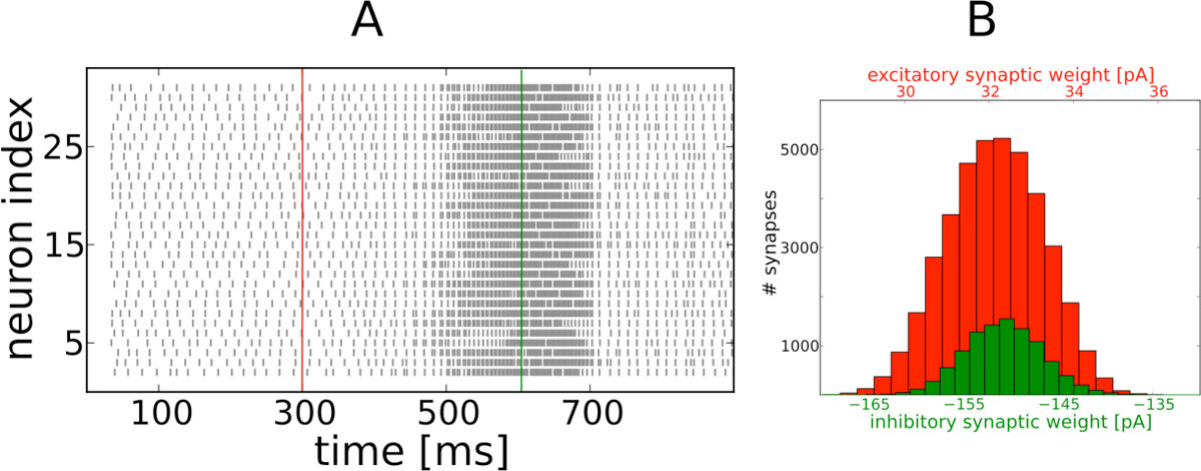
**A. Raster plot of 30 randomly sampled cells showing network activity**. Red line: activation of excitatory STDP, green line: activation of inhibitory STDP. **B**. Weight distributions of plastic synapses converging onto 100 randomly sampled excitatory neurons. Red: excitatory synapses, green inhibitory synapses.

## References

[B1] Vogels TP Sprekeler H Zenke F Clopath C Gerstner W Inhibitory plasticity balances excitation and inhibition in sensory pathways and memory networks Science 2011 334 1569 73 (New York, NY) 10.1126/science.1211095 22075724

[B2] Brunel N Dynamics of sparsely connected networks of excitatory and inhibitory spiking neurons Journal of computational neuroscience 2000 8 183 208 10.1023/A:1008925309027 10809012

[B3] Morrison A Aertsen A Diesmann M Spike-timing-dependent plasticity in balanced random networks Neural computation 2007 19 1437 67 10.1162/neco.2007.19.6.1437 17444756

[B4] Litwin-Kumar A Doiron B Slow dynamics and high variability in balanced cortical networks with clustered connections Nature Neuroscience 2012 15 1498 1505 10.1038/nn.3220 23001062 PMC4106684

